# Moderately overweight and obese patients in general practice: a population based survey

**DOI:** 10.1186/1471-2296-7-43

**Published:** 2006-07-07

**Authors:** Liset van Dijk, Hanneke B Otters, Albertine J Schuit

**Affiliations:** 1 NIVEL, Netherlands Institute for Health Services Research, P.O. Box 1568, 3500 BN Utrecht, The Netherlands; 2 Department of General Practice, Erasmus MC, University Medical Centre Rotterdam, P.O. Box 1738, 3000 DR Rotterdam, The Netherlands; 3 National Institute for Public Health and the Environment, P.O. Box 1, 3720 BA Bilthoven, The Netherlands; 4 Institute of Health Science, Free University, Amsterdam, The Netherlands

## Abstract

**Background:**

Obesity is a main threat to public health in the Western world and is associated with diseases such as diabetes mellitus and coronary heart diseases. Up to now a minority of research studied the relation between obesity and the use of primary health care. In the Netherlands the general practitioner (GP) is the main primary health care provider. The objective of this article is to evaluate GP consultation and prescription of drugs in moderate and severely overweight (obese) persons in the Netherlands.

**Methods:**

Data were used from a representative survey of morbidity in Dutch general practice in 2001. Our study sample consisted of 8,944 adult respondents (18+ years) who participated in an extensive health interview. Interview data were linked to morbidity and prescription registration data from 95 general practices where respondents were listed. Body mass index (BMI) was calculated using self-reported height and weight. Analyses were controlled for clustering within practices as well as for socio-demographic and life style characteristics.

**Results:**

Obesity (BMI ≥ 30 kg/m2) was observed in 8.9% of men and 12.4% of women; for moderate overweight (BMI 25-<30 kg/m2) these percentages were 42.2% and 30.4% respectively. Obese men and women were more likely to consult their GP than persons without overweight. This especially holds for diseases of the endocrine system, the cardiovascular system, the musculoskeletal system, the gastro-intestinal system, and skin problems. Related to this, obese men and women were more likely to receive drugs for the cardiovascular system, the musculoskeletal system, alimentary tract and metabolism (including, for example, antidiabetics), and dermatologicals, but also antibiotics and drugs for the respiratory system. For moderately overweight men and women (BMI 25-<30 kg/m2) smaller but significant differences were found for diseases of the endocrine system, the cardiovascular system, and the musculoskeletal system.

**Conclusion:**

Obesity increases the workload of Dutch general practitioners and the use of prescribed medication. The current increase in the prevalence of obesity will further increase the use of health care and related costs. Since a large majority of Dutch persons visit their GP over the course of one year, GPs' potential role in effective prevention strategies cannot be denied.

## Background

Obesity is one of the main threats to public health in the Western world [1,2]. It is a well-known risk factor for coronary heart diseases and type 2 diabetes mellitus [e.g. [3-10]]. Other health problems such as musculoskeletal disorders, psychological problems, osteoarthritis, cancer, and respiratory problems have been related to obesity as well [11-17]. Obesity decreases life expectancy with almost seven years [18] and obese persons have an impaired quality of life [19]. Costs of obesity in various countries have been estimated at 4 to 8% of total health care costs [20-24]. In the Netherlands about 40% of the population is overweight (BMI > 25 kg/m2) and about 10% of the total population is obese (BMI ≥ 30 kg/m2) [25]. Although the prevalence of obesity in the Netherlands is still lower than in most other European countries [2,26] and the USA [27], an increase has been observed over the last decade [28] This trend is worrying and prompted the National Health Council of the Netherlands to urge for further research with respect to assessing trends and examining effective prevention strategies [28].

Up to now, a minority of studies focused on the association between obesity and the use of primary care. In Anglo-Saxon countries, where the problem of obesity is even more abound than in the Netherlands, it was found that obesity increased the use of primary health care as well as the use of prescribed medication [24,29,30]. These studies, however, did not include information on persons with modest overweight. Moreover, they did not take into account other life style factors such as smoking habits, exercising, and alcohol consumption. In the Netherlands, extra need for health care caused by obesity and overweight will primarily be handled by general practitioners. Dutch GPs have an important role within the health care system since they perform a gatekeeper role. All Dutch inhabitants are listed with a general practice and the GP is the first one to be consulted for health problems. Dutch GPs deal with 96% of all problems themselves; only in 4% of their consultations GPs refer their patients to a medical specialist or to other primary care providers [31]. Prescribing drugs is the most common treatment in Dutch general practice [31,32] and it generates considerable cost: almost € 3.8 billion in 2001 [33]. GPs prescribe the majority of medication in the Netherlands [34].

It is not clear what the extra need for GP care and associated treatment among obese and moderately overweight patients in the Netherlands is. In this study we compare GP consultation and drug prescribing in obese and moderately overweight Dutch adults (18+ years) with GP consultation and drug prescribing in Dutch adults without overweight taking into account socio-demographic characteristics and other life style factors.

## Methods

Data were used from the Second Dutch National Survey of General Practice in 2001 [35]. This nationwide survey was conducted by the Netherlands Institute for Health Services Research (NIVEL) in cooperation with the National Institute for Public Health. At first, practices already participating in the Dutch National Information Network of General Practice (LINH) were invited to participate in this study because of their experience in the use of electronic medical records [36]. Sixty-one LINH-practices agreed to participate. To recruit more practices a mailing was sent out to a sample of GPs stratified with respect to type of practice, level of urbanisation, and region. Stratification was used to increase the representativeness of the participants. Another 43 practices were willing to participate. The 104 general practices compromised 195 GPs. The participating GPs were representative for the Dutch GP population on age, sex, part-time/fulltime working, urbanisation level, and region. Single-handed practices were under-represented.

In the Netherlands, all inhabitants are listed in a general practice. The patient population of the 104 practices was representative for the Dutch population as a whole [35,36]. Approximately 5% of the Dutch-speaking listed patients in the 104 practices were asked to participate in an extensive health interview (n = 19,685). A letter from the GP accompanied the invitations. Selection for the interview was random, with a fixed target per full-time GP. The response rate to the interview was 64.5% and the total number of respondents was 12,699; 9,685 respondents were 18 years or older. The distribution of the respondents according to age, gender and residence was comparable to that of the Dutch population [35]. Privacy of the participating persons was guaranteed and in accordance with Dutch legislation. Patients were informed about the study prior to the start of the data collection via a personal letter written by their GP and by announcements in the practice [35].

Trained interviewers interviewed respondents at home. The interviews were evenly distributed among four consecutive 3-months periods to correct for seasonal influences. Items used for this study included self-reported weight and height, socio-economic status, and life style characteristics. Interview data of the respondents were linked to their morbidity and prescription data as registered by their GP in 2001. Over the course of one year, GPs who participated in the survey registered all contacts with their patients. This included face-to-face consultations as well as telephone consultations and repeat prescriptions. Every single health problem presented within a consultation was coded by the GP using the International Classification for Primary Care (ICPC) [37]. When a patient presented two complaints within one consultation, these were coded as two separate (sub)consultations. Different consultations for the same health problem were clustered into disease episodes. Medical students who were trained by NIVEL staff members did this after the data collection. GPs also registered all prescriptions using the Anatomical Therapeutical Chemical (ATC) coding system [38]. Therefore it is known for every patient how often he or she contacted the GP and for which health problems. Moreover, it was known how many prescriptions the patient received and what type of medication it was. Patients from nine practices were excluded because of insufficient quality of the morbidity and/or prescription registration data (n = 543 patients). Another 198 respondents were excluded because of incomplete data on weight and/or height or a BMI of < 15 kg/m2. Included in the analyses were 8,944 respondents with complete data (men: 4,048; women: 4,896).

As a measure for overweight and obesity, body mass index (BMI) was used. BMI was based on self-reported weight and height. Three groups were distinguished: without overweight (BMI < 25 kg/m2), moderately overweight (BMI 25- < 30 kg/m2), and obese (≥ 30 kg/m2). GP consultation was assessed in different ways. The first measure was whether or not the patient visited the GP during the year of registration. Moreover, the number of consultations with the GP was assessed. As stated before, every consultation was coded according to the ICPC. This classification contains different chapters, each referring to a group of diseases. When a patient consulted the GP for hypertension, this patient was coded as having consulted the GP for a complaint/disease in ICPC-chapter K (cardiovascular diseases).

The assessment of drug prescribing was comparable to that of GP consultation. The first assessment referred to the question whether or not the patient received at least one prescription in 2001. The number of prescriptions per patient was also assessed. The ATC-classification system distinguishes drugs for different diseases. For each ATC-chapter it was assessed whether the patient got at least one prescription (versus none) within the respective chapter.

Since both obesity and morbidity are associated with lower socio-economic status, age, and lifestyle [39], analyses were controlled for these factors. Included variables were: age, type of health insurance (private/public), place of residence (urban/rural), educational level (low/middle/high), smoking (current smoker/non-smoker), alcohol consumption (no alcohol consumption/1–2 glasses per day/more than 2 glasses per day) and number of days per week that respondents spends at least 30 minutes in exercise activities such as walking, cycling, and gardening (never/1 to 3 days per week/4 to 6 days per week/every day).

Three categories for BMI were distinguished in all analyses: without overweight (BMI < 25 kg/m2), moderately overweight (BMI 25- <30 kg/m2), and obese (≥ 30 kg/m2). Moreover, men and women were analysed separately. We compared socio-demographics and life style characteristics for the three BMI-categories. Differences between BMI-categories were tested with chi-square tests. Chi-square tests were also used to test differences between BMI-categories in the proportion of respondents who consulted their GP and the proportion of respondents who received a prescription. We used multilevel logistic regression to calculate the odds of visiting the GP for a certain ICPC-code. Reference category in these analyses was the BMI-category "no overweight". The multilevel design was chosen because of the nested structure of the data – our patients were sampled from general practices -. It was also used to assess differences between BMI-categories in drug prescribing for specific ATC-chapters. All multilevel logistic regressions were controlled for sociodemographic and life style variables.

## Results

### Prevalence of obesity and overweight

Obesity was reported by 8.9% of male and 12.4% of female respondents (Table 1). Men were more often moderately overweight than women: 42.2% and 30.4% respectively. Both male and female respondents with moderate overweight and obesity were less well educated, more often had public health insurance, spent less time in exercising, but had a lower alcohol consumption compared to respondents without overweight. Especially in the age category 55–74 years women more often were obese, while for men the age category 35–54 years was overrepresented among the obese group as well. Overweight and obese women smoked less often than women without overweight.

**Table 1:** Distributions of sociodemographic and life style characteristics among BMI-categories; men and women (N = 8,944)a)

	Men				Women			
	BMI < 25 kg/m^2 ^(n = 1980)	BMI 25- <30 kg/m^2 ^(n = 1707)	BMI = 30 kg/m^2 ^(n = 361)		BMI < 25 kg/m^2 ^(n = 2800)	BMI 25- <30 kg/m^2 ^(n = 1488)	BMI = 30 kg/m^2 ^(n = 608)	

Age				p < 0.001				p < 0.001
< 35 years	31.5	14.2	13.0		28.9	13.1	12.5	
35–54	40.3	46.3	51.5		44.9	43.3	43.6	
55–74	21.6	33.0	31.3		19.5	33.4	36.0	
75plus	6.6	6.4	4.2		6.8	10.2	7.9	
								
Educational level				p < 0.001				p < 0.001
Low	28.8	35.9	46.0		28.5	44.8	55.9	
Middle	45.0	41.4	40.7		48.1	40.4	34.2	
High	26.2	22.7	13.3		23.3	14.7	9.9	
								
Health insurance				p = 0.005				p < 0.001
Public	58.8	58.2	67.3		68.8	73.9	79.8	
Private	41.2	41.8	32.7		31.2	26.1	20.2	
								
Place of residence				p = 0.008				ns
Urban	35.1	30.2	33.5		35.9	33.6	34.9	
Rural	64.9	69.8	66.5		64.1	66.4	65.1	
								
Smoking				p = 0.001				p < 0.001
Non-smoker	60.9	66.9	64.0		70.8	75.9	76.6	
Current smoker	39.1	33.1	36.0		29.2	24.1	23.4	
								
Alcohol consumption				p < 0.001				p < 0.001
None	14.8	17.5	24.3		28.2	37.0	45.0	
s 1–2 glass p/day	58.8	57.8	51.7		64.5	56.4	50.8	
> 2 glasses p/day	26.4	24.7	24.0		7.3	6.6	4.2	
								
Exercise (> 30 minutes p/day)				p = 0.034				p < 0.001
< 1 day p/week	10.2	10.5	15.6		12.5	17.5	21.6	
1–3 days p/week	24.7	26.8	25.6		21.0	19.5	21.8	
4–6 days p/week	24.2	22.0	22.8		21.6	20.8	20.6	
all days	40.8	40.8	36.1		45.0	42.2	36.0	
								
% of all men/women	48.9	42.2	8.9	100%	57.2	30.4	12.4	100%

a) significance test: Pearson Chi-square test, ns = not significant (p > 0.05)

### GP consultation

**Figure 1:**
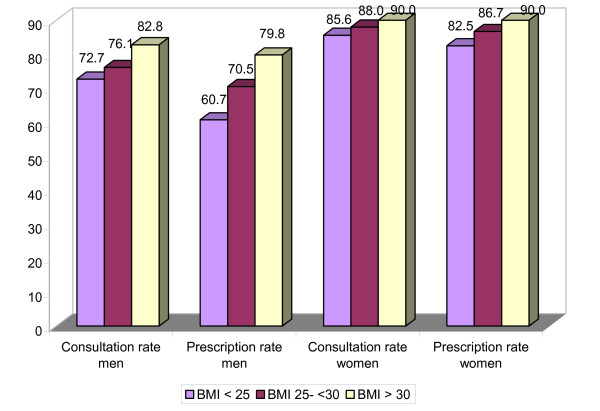
Percentage of men and women consulting their GP and receiving a prescription according to BMI (n = 8,944)^a)^.

The percentage of respondents who consulted their GP at least once in 2001 was highest among obese respondents and lowest among respondents with BMI < 25 kg/m2. While 82.2% of obese men consulted their GP in 2001, 72.7% of men without overweight did so (Figure 1). For women the difference was smaller: 90% of obese women and 85.6% of women without overweight contacted their GP in 2001.

**Figure 2:**
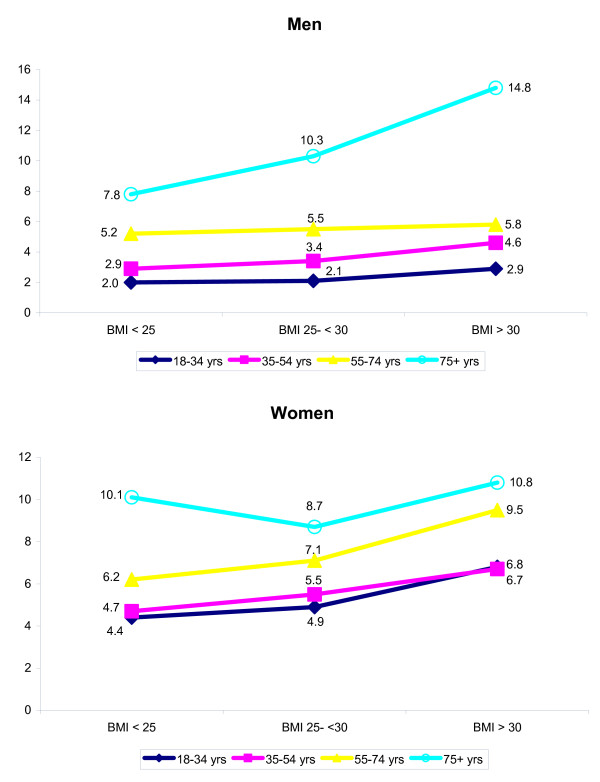
Average number of visits to GP by age and BMI in 2001 (N = 8,944).

The number of visits to the GP in 2001 was higher in obese men compared to men without overweight (Figure 2). The difference was highest among the eldest: obese men in this age category visited their GP twice as often compared to their peers without overweight. Obese women in all age categories, except the eldest, consulted their GP more often than women without overweight.

Table 2 and Table 3 show that, for both men and women, differences in GP consultation can be attributed to some categories of diseases in particular. Differences in consultation rates between respondents without overweight and obese respondents were most profound for diseases of the endocrine system, the cardiovascular system, the musculoskeletal system, the gastro-intestinal system, skin problems and, in women, diseases of the respiratory system. These differences remained significant after controlling for sociodemographic characteristics and life style factors. For diseases of the cardiovascular and endocrine system differences between obese and non-overweight respondents were larger in men than in women.

**Table 2:** Differences in GP consultation of men in different BMI-categories: bivariate and multilevel analyses

	Bivariate analyses ^a) ^Percentage that visited GP for ICPC-chapter				Multilevel logistic regression ^b,c) ^Adjusted odds ratios [95% BI]			
ICPC-Chapter	BMI < 25 kg/m^2 ^(n = 1980)	BMI 25- <30 kg/m^2 ^(n = 1707)	BMI = 30 kg/m^2 ^(n = 361)		BMI 25- <30 kg/m^2^		BMI ≥ 30 kg/m^2^	

General (A)	8.8	10.5	10.2	ns	1.18	[0.94–1.48]	1.08	[0.74–1.59]
Blood and blood forming organs (B)	2.0	1.5	1.9	ns	0.63	[0.38–1.05]	0.76	[0.33–1.75]
Gastro-intestinal (D)	13.2	15.0	19.7	p = 0.004	1.01	[0.84–1.23]	1.37*	[1.02–1.84]
Eye (F)	7.0	7.1	7.5	ns	0.92	[0.71–1.19]	0.93	[0.60–1.44]
Ear (H)	8.9	11.5	10.8	ns	1.17	[0.94–1.45]	1,08	[0.74–1.56]
Cardiovascular system (K)	13.9	21.1	31.6	p < 0.001	1.32*	[1.08–1.61]	2.90*	[2.16–3.88]
Musculoskeletal system (L)	28.0	34.9	38.5	p < 0.001	1.32*	[1.14–1.52]	1.46*	[1.15–1.86]
Nervous system (N)	5.1	6.5	8.0	p = 0.043	1.22	[0.92–1.62]	1.45	[0.61–3.47]
Psychic problems (P)	8.9	9.5	11.6	ns	1.09	[0.87–1.38]	1.33	[0.92–1.92]
Respiratory system (R)	24.7	27.3	31.6	p = 0.014	1.00	[0.85–1.16]	1.18	[0.91–1.52]
Skin (S)	25.5	27.7	32.7	p = 0.014	1.07	[0.92–1.24]	1.30*	[1.02–1.67]
Endocrine system (T)	5.8	11.7	19.7	p < 0.001	1.79*	[1.40–2.30]	3.44*	[2.45–4.82]
Urinary tract (U)	3.7	4.2	3.9	ns	0.91	[0.65–1.29]	0.79	[0.42–1.46]
Reproductive system men (Y)	6.6	7.0	6.1	ns	0.91	[0.70–1.18]	0.80	[0.49–1.28]
SSocial problems (Z)	2.0	2.2	1.4	ns	1.08	[0.67–1.73]	0.61	[0.24–1.58]

a) significance test: Pearson Chi-square test, ns = not significant (p < 0.05), ns = not significant (p > 0.05)

b) Multilevel logistic regression, odds ratios are corrected for age, sex, educational level, type of health insurance, residence, smoking, alcohol consumption and exercising

c) reference in multilevel logistic regression is BMI < 25

* p < 0.05

**Table 3:** Differences in GP consultation of women in different BMI-categories: bivariate and multilevel analyses

	Bivariate analyses ^a) ^Percentage that visited GP for ICPC-chapter				Multilevel logistic regression ^b,c) ^Adjusted odds ratios [95% BI]			
ICPC-Chapter	BMI < 25 kg/m^2 ^(n = 2800)	BMI 25- <30 kg/m^2 ^(n = 1488)	BMI = 30 kg/m^2 ^(n = 608)		BMI 25- <30 kg/m^2^		BMI ≥ 30 kg/m^2^	

General (A)	14.0	14.0	16.0	ns	0.92	[0.76–1.11]	1.03	[0.80–1.33]
Blood and blood forming organs (B)	4.0	3.7	4.1	ns	0.85	[0.60–1.19]	0.86	[0.54–1.37]
Gastro-intestinal (D)	15.7	17.5	23.4	p < 0.001	0.99	[0.83–1.18]	1.35*	[1.08–1.69]
Eye (F)	8.2	10.1	10.0	ns	0.99	[0.79–1.24]	1.02	[0.75–1.39]
Ear (H)	10.6	11.6	12.3	p = 0.031	0.96	[0.78–1.18]	1.03	[0.78–1.36]
Cardiovascular system (K)	15.4	26.3	35.4	p < 0.001	1.37*	[1.15–1.62]	2.16*	[1.73–2.69]
Musculoskeletal system (L)	37.1	44.8	51.3	p < 0.001	1.22*	[1.06–1.39]	1.52*	[1.25–1.84]
Nervous system (N)	9.6	11.4	13.2	p = 0.017	1.08	[0.86–1.32]	1.20	[0.91–1.58]
Psychic problems (P)	15.3	16.5	15.5	ns	0.96	[0.80–1.15]	0.83	[0.64–1.07]
Respiratory system (R)	29.9	34.7	42.1	p < 0.001	1.08	[0.94–1.24]	1.41*	[1.17–1.71]
Skin (S)	29.9	30.0	36.2	p = 0.007	0.99	[0.86–1.15]	1.25*	[1.03–1.52]
Endocrine system (T)	5.6	11.7	17.9	p < 0.001	1.62*	[1.28–2.05]	2.52*	[1.91–3.33]
Urinary tract (U)	9.4	11.1	13.7	p = 0.005	1.00	[0.81–1.23]	1.19	[0.90–1.57]
Pregnancy, anticonception (W)	20.2	12.2	10.0	p < 0.001	1.09	[0.88–1.35]	0.81	[0.58–1.14]
Reproductive system women (X)	25.9	21.6	28.6	p = 0.003	0.93	[0.79–1.09]	1.19	[0.96–1.47]
Social problems (Z)	4.0	3.0	4.3	ns	0.70	[0.49–1.02]	1.00	[0.64–1.58]

a) significance test: Pearson Chi-square test, ns = not significant (p < 0.05), ns = not significant (p > 0.05)

b) Multilevel logistic regression, odds ratios are corrected for age, sex, educational level, type of health insurance, residence, smoking, alcohol consumption and exercising

c) reference in multilevel logistic regression is BMI < 25

* p < 0.05

Moderately overweight men and women (BMI 25- <30 kg/m2) were more likely to consult their GP for cardiovascular problems as well as musculoskeletal problems and diseases of the endocrine system than non-overweight persons but less likely than obese respondents.

### Drug prescribing

**Figure 3:**
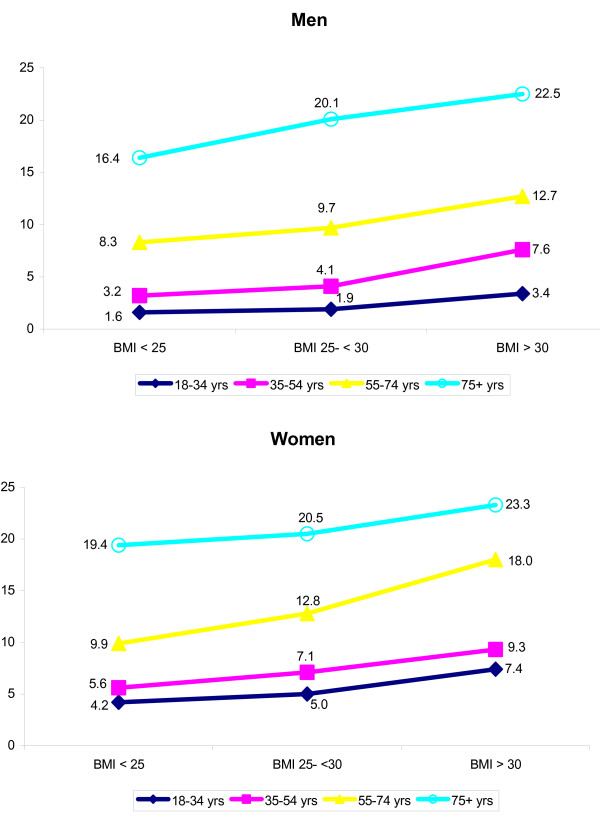
Average number of prescriptions received from GP by age and BMI (N = 8,944).

The percentage of respondents who received a prescription in 2001 was highest among obese respondents and lowest among those without overweight. Differences were larger for men than for women (Figure 1). Considerable differences existed between the average numbers of prescriptions for the three BMI-categories (Figure 3), although there were some differences between men and women and for different age categories. The largest difference was found in women aged 55–74 years. Non-overweight women in this age category received 9.9 prescriptions while their obese peers received almost twice as many prescriptions: 18.0.

In almost all ATC-categories a significant difference between the BMI-categories was observed in the percentage of respondents who received a prescription (Table 4 and Table 5). After controlling for socio-demographic and life style characteristics it proved that GPs prescribed more often drugs for the cardiovascular system, drugs for the musculoskeletal system, drugs for the alimentary tract and metabolism (including for example antidiabetics), and dermatologicals to obese men and women than to non-overweigh respondents. The prescription of these drugs is related to the diseases for which obese respondents had a higher probability of consulting their GP. Obese respondents were more likely to receive prescriptions for antibiotics and drugs for the respiratory system as well.

**Table 4:** Differences in prescribed drugs for men in different BMI-categories: bivariate and multilevel analyses

	Bivariate analyses ^a) ^Percentage that got prescribed for ICPC-chapter				Multilevel logistic regression ^b,c)^ Adjusted odds ratios [95% BI]			
ATC-Chapter	BMI < 25kg/m^2^ (n = 1980)	BMI 25- <30 kg/m^2^ (n = 1707)	BMI = 30 kg/m^2^ (n = 361)		BMI 25- <30 kg/m^2^		BMI ≥ 30 kg/m^2^	

Alimentary tract and metabolism (A)	14.0	16.9	25.2	p < 0.001	1.01	[0.83–1.22]	1.66*	[1.24–2.21]
Blood and blood forming organs (B)	9.2	14.2	13.3	p < 0.001	1.29*	[1.02–1.63]	1.23	[0.83–1.81]
Cardiovascular system (C)	14.6	25.2	32.1	p < 0.001	1.62*	[1.34–1.97]	2.81*	[2.09–3.77]
Dermatologicals (D)	18.9	22.1	26.3	p = 0.002	1.12	[0.95–1.32]	1.37*	[1.05–1.80]
Genito urinary system and sex homones (G)	3.3	4.5	3.3	ns	1.11	[0.78–1.58]	0.84	[0.43–1.64]
Systemic hormonal preparations (H)	3.7	4.5	8.0	p = 0.001	1.01	[0.73–1.42]	1.79*	[1.13–2.85]
Antiinfectives for systemic use (J)	21.6	27.5	32.4	p < 0.001	1.12	[0.95–1.31]	1.36*	[1.04–1.77]
Antineoplastic and immunomodulating agents (L)	0.4	0.4	0.6	ns	0.95	[0.33–2.75]	1.20	[0.24–5.98]
Musculoskeletal system (M)	14.3	20.8	30.2	p < 0.001	1.43*	[1.20–1.71]	2.18*	[1.67–2.85]
Nervous system (N)	14.8	18.2	21.6	p = 0.001	1.12	[0.94–1.35]	1.30	[0.97–1.74]
Antiparasitic products (P)	0.8	0.8	1.7	ns	0.96	[0.45–2.01]	2.16	[0.82–5.64]
Respiratory system (R)	15.8	18.6	23.0	p < 0.001	1.12	[0.94–1.33]	1.40*	[1.06–1.85]
Sensory organs (S)	7.9	8.6	9.4	ns	0.98	[0.77–1.25]	1.01	[0.67–1.51]

a) significance test: Pearson Chi-square test, ns= not significant (p < 0.05), ns = not significant (p > 0.05)

b) Multilevel logistic regression, odds ratios are corrected for age, sex, educational level, type of health insurance, residence, smoking, alcohol consumption and exercising

c) reference in multilevel logistic regression is BMI < 25

* p < 0.05

**Table 5:** Differences in prescribed drugs for women in different BMI-categories: bivariate and multilevel analyses

	Bivariate analyses ^a) ^Percentage that got prescribed for ICPC-chapter				Multilevel logistic regression ^b,c)^ Adjusted odds ratios [95% BI]			
ATC-Chapter	BMI < 25 kg/m^2^ (n = 1980)	BMI 25- <30 kg/m^2^ (n = 1707)	BMI = 30 kg/m^2^ (n = 361)		BMI 25- <30 kg/m^2^		BMI ≥ 30 kg/m^2^	

Alimentary tract and metabolism (A)	16.7	23.4	30.4	p < 0.001	1.13	[0.96–1.34]	1.53*	[1.24–1.90]
Blood and blood forming organs (B)	9.0	11.7	15.5	p < 0.001	0.90	[0.72–1.12]	1.16	[0.88–1.54]
Cardiovascular system (C)	15.1	28.8	37.8	p < 0.001	1.58*	[1.33–1.88]	2.53*	[2.02–3.16]
Dermatologicals (D)	23.4	24.5	32.4	p < 0.001	1.00	[0.85–1.16]	1.45*	[1.18–1.77]
Genito urinary system and sex homones (G)	37.9	28.7	26.5	p < 0.001	1.03	[0.88–1.21]	0.87	[0.70–1.09]
Systemic hormonal preparations (H)	5.4	8.4	10.7	p < 0.001	1.23	[0.95–1.58]	1.53*	[1.12–2.11]
Antiinfectives for systemic use (J)	28.9	36.9	42.1	p < 0.001	1.11	[0.96–1.28]	1.35*	[1.12–1.64]
Antineoplastic and immunomodulating agents (L)	0.8	0.9	1.5	ns	0.97	[0.48–1.97]	1.61	[0.72–3.60]
Musculoskeletal system (M)	20.6	29.6	36.0	p < 0.001	1.38*	[1.18–1.60]	1.75*	[1.43–2.14]
Nervous system (N)	26.1	33.1	35.0	p < 0.001	1.09	[0.94–1.26]	1.15	[0.94–1.40]
Antiparasitic products (P)	1.5	0.9	1.5	ns	0.72	[0.39–1.35]	0.94	[0.41–2.16]
Respiratory system (R)	20.9	25.1	31.1	p < 0.001	1.23*	[1.05–1.44]	1.62*	[1.33–1.99]
Sensory organs (S)	9.7	11.6	11.8	ns	1.04	[0.85–1.29]	1.03	[0.78–1.38]

a) significance test: Pearson Chi-square test, ns= not significant (p < 0.05), ns = not significant (p > 0.05)

b) Multilevel logistic regression, odds ratios are corrected for age, sex, educational level, type of health insurance, residence, smoking, alcohol consumption and exercising

c) reference in logistic regression is BMI < 25

* p < 0.05

Moderately overweight men and women (BMI 25- <30 kg/m2) were more likely to get prescribed drugs for the cardiovascular system and for the musculoskeletal system. Moreover, moderately overweight men more often received drugs for blood and blood forming organs compared to men without overweight, while moderately overweight women received more prescriptions for drugs of the respiratory system than non-overweight women.

## Discussion

### Comparison with previous research

The results of this cross-sectional study show that obesity increases the workload of Dutch general practitioners. Independent of differences in gender, age, social status and life style, obese Dutch persons are more likely to consult their GP and need pharmacotherapeutical care more often. This was also found in countries with a higher proportion of obese inhabitants than the Netherlands: the UK and the US [24,29,30]. Differences in GP consultation are largest for diseases of the endocrine system, the cardiovascular system and the musculoskeletal system, which is in line with findings of previous epidemiological research on the positive association between obesity and cardiovascular diseases, type 2 diabetes mellitus and osteoarthritis (as described in the introductory part of this contribution). Moreover, obese persons were more likely to consult their GP for skin problems and gastro-intestinal problems. Drug prescribing was largely related to the morbidity pattern, as was also found in a study published by the Counterweight Team [24]. However, obese persons were more likely to use antibiotics and drugs for respiratory system, while they did not consult the GP more often for respiratory problems. For this latter group of drugs, it may well be that obese persons consulted the medical specialist, but received their (repeat) prescriptions from their GP. Obese persons were not likely to present more psychological problems to their GP, while it has been suggested that psychological problems and obesity are associated [28]. Earlier research on the impact of obesity on the use of primary health care did not include persons with moderate overweight (BMI 25- < 30 kg/m^2^) [24,29,30]. Moderately overweight persons consulted their GP more often for diseases of the endocrine system, cardiovascular diseases and musculoskeletal problems and got more prescriptions related to these diseases than persons without overweight, albeit differences with non-overweight persons were clearly smaller than for obese persons. It should be noted that we included persons (n = 173) with low BMI (< 18.5 kg/m^2^) in our reference category. Albeit these persons tend to consult their GP more often, additional bivariate and multivariate analyses showed that there was hardly any significant difference between them and persons with BMI 18.5–25 kg/m^2 ^. Previous studies in this field did not assess men and women separately [24,29,30]. Our results showed that there are some differences between men and women, but these differences are not striking.

### Strengths and limitations

This study used cross-sectional data from almost 10,000 Dutch adult persons who were listed in 95 general practices. Data from a health interview were linked to data from their GP's registration. The combination of such extensive interview data combined with GP registration data is unique. The extensive interview data made it possible to control the analyses for a large set of possible confounders such as smoking, alcohol consumption, and exercising. Moreover, clustering within general practices was taken into account in the multilevel analyses. Contrary to the Counterweight Project [24,30] our study was not specifically developed to study the impact of obesity on health care use. Therefore, it is not plausible to assume a bias in the participation of general practitioners with regard to the topic of our study. However, patients' participation in the health interview may be biased. There was a 34.5 percent non-response to the health interview. It has been found that non-respondents more often have poorer health [40]. Therefore, it may be well that obese and overweight persons more often refused to participate in the interview.

Another limitation of the study is the use of self-reported data on weight and height. A recent study by Ezzati et al showed that women tend to underreport their weight [41]. Men did not, but young and middle-aged men over-report their height. Both phenomena lead to an underestimation of BMI. This would imply that the impact of weight on the use of GP care and on drug prescribing is underestimated in our study. However, Ezzati et al also showed that the bias was larger in telephone interviews compared to in-person interviews. Such face-to-face interviews clearly promote the validity of answers on self-reported height and weight. The proportions of overweight and obese persons we found in our study were almost comparable to those of Dutch studies where weight and height were actually measured by the study personnel [28].

### Clinical implications

Almost all obese and moderately overweight persons consult their GP at least once a year. Because of these high consultation rates, GPs' potential role in weight management, the prevention of complications of obesity, and related use of health care is strong. Moreover, it can be assumed that advice to lose weight is more effective if linked to patients' health problems such as coronary heart disease, musculoskeletal problems and diabetes mellitus. However, up to now weight management is not embedded in general practice. British researchers argued that obesity is under-recognized in primary care [42]. There is no reason to assume that this is different in the Netherlands. Attempts have been made to improve weight management in primary care in the UK, with some attempts being more successful than others [43-45]. As such, there still seems to be need for good weight management systems in general practice and for methods with long-term effects. First of all, it seems important that GP acknowledge their role in weight management more than they currently do. GPs tend to conceptualise obesity in terms of responsibility and they consider weight management to be the patient's responsibility [46]. As such GPs do not think that weight management is within their professional domain. However, given the results of the present and other studies [24,29,30,47] it would be worthwhile if GPs should become aware of the important role they potentially can have in weight management and related health care use. This is especially true for countries like the Netherlands where GPs function as gatekeepers and usually are the first professionals confronted with health care problems of their patients.

## Conclusion

Obesity and, to a lesser extent, moderate overweight, increase the workload of Dutch general practitioners and the use of prescribed medication. The current expansion in the prevalence of obesity will further increase the use of health care and related costs, especially for diseases of the endocrine system, the cardiovascular system, and the musculoskeletal system. This prompted the National Health Council of the Netherlands to urge for further examination of effective prevention strategies in this area. Since a large majority of all Dutch persons visit their GP over the course of a year, GPs' potential role in prevention strategies cannot be denied.

## Competing interests

The author(s) declare that they have no competing interests.

## Authors' contributions

LvD formulated the research question, designed and managed the study, drafted and revised the manuscript. HO and AS advised on the analyses and critically reviewed draft versions of the manuscript. All authors read and approved the final manuscript.

## Pre-publication history

The pre-publication history for this paper can be accessed here:


